# Genome editing: Bioethics shows the way

**DOI:** 10.1371/journal.pbio.2001934

**Published:** 2017-03-16

**Authors:** Carolyn P. Neuhaus, Arthur L. Caplan

**Affiliations:** Division of Medical Ethics, NYU School of Medicine, New York, New York, United States of America

## Abstract

When some scientists hear the word “bioethics,” they break out in intellectual hives. They shouldn’t. Good bioethics is about enabling science to move forward. Bioethics pushes scientists to acknowledge that they operate not within a vacuum but within a society in which diverse perspectives and values must be engaged. Bioethicists give voice to those divergent perspectives and provide a framework to facilitate informed and inclusive discussions that spur progress, rather than stall it. The field is needed to advance cutting-edge biomedical research in domains in which the benefits to be had are enormous, such as genome editing, but ethical concerns persist.

When some scientists hear the word “bioethics,” they break out in intellectual hives. They shouldn’t. Bioethics has done far more good than harm in advancing medical research: creating a framework for responsible research with animals, involving vulnerable and marginalized persons in clinical research, and providing a moral foundation for transplant research. Perhaps the same cannot be said for lawyers, regulators, politicians, or theologians, but they do not represent bioethics.

Bioethicists think about the ethical aspects of biomedical research and its consequences. The best bioethics work sparks conversations inside research communities and then moves them into the public sphere. Sometimes bioethics research exposes unethical practices in science and medicine, such as historical abuses of human subjects in the Tuskegee syphilis experiments [1]. Other times, bioethicists think prospectively about the ethics of emerging technologies. Workable solutions to past and current ethical concerns often emerge despite disagreements. Good bioethics is about enabling science to move forward rather than raising simpleminded in-principle objections, vilifying researchers, invoking arguments against playing the divinity, or setting arbitrary limits. The field is needed to advance cutting-edge biomedical research in domains in which the benefits to be had are enormous, such as genome editing, but ethical concerns persist.

For example, the discovery of recombinant DNA (rDNA) in the 1970s ignited discussions around the ethics of eugenics and using the technology to intentionally modify the human genome to create designer babies. The fact that these terms require no clarifying definitions means that bioethicists have largely been successful at promoting public discourse about what it would mean to design humans. We haven’t achieved consensus over whether we should select the traits of our offspring, but an international conversation is ongoing about the issue. Since the initial ethical discussions surrounding rDNA, scientists have mapped the human genome, rolled out gene therapies, and developed improved genome-editing techniques. That’s bioethical success: cautiously moving forward while staying informed about the ethical issues and remaining sensitive to a wide diversity of perspectives.

Other normative questions raised by advances in genetics have gotten fewer journal pages but are equally important to advancing science. Consider genome editing in nonhuman organisms. While ethical debate tends to focus on human applications, issues around the use of genome editing in animals require attention and may well help clarify the limits and boundaries of human use.

Genome editing makes possible large-scale production of disease models in large animals, like pigs, dogs, and nonhuman primates. While the creation of model organisms sometimes reveals new insights into the genetic underpinnings of disease and facilitates characterization of genetic pathways, it also contributes to animal suffering, perpetuates the use of animals in research, and challenges fundamental assumptions about the moral status of animals. Yet, research will proceed if suffering is minimized, utility and safety established, and boundaries put on what can be done to animals in the pursuit of knowledge (as opposed to the pursuit of amusement).

Or, consider the use of genome editing to eliminate pest populations and disease vectors in the wild, like invasive species, mosquitoes, or mice. Such applications promise to eliminate some of the globe’s biggest public health threats but could also disrupt ecosystems. Public pushback against genetically modified food does not bode well for the prospects of widespread use of either genetically modified insects or animals. But, even despite a fraught history, the future of genetic engineering to fight blights, fungi, and insect pests is not doomed.

If you and your neighbor disagree over whether to release genetically modified mosquitoes in your community as part of a research experiment, should the experiment go on? Should branding and fail-safe mechanisms such as terminator genes be in place before a genetically modified animal or insect is released into the wild? A prudent path forward for ethics research on gene drives and genetic solutions to environmental challenges will create forums for inclusive public discussions and integrate divergent perspectives into ethics literature and policy. Proposals for how to do this through community engagement can be found in Massachusetts [2] and the Florida Keys [3].

Consider the rapid development of gene therapies, often combined with stem cell modifications, to combat cancer. When are gene therapies ready for first-in-human use? The death of five patients in a 2016 clinical trial of chimeric antigen receptor T cell (CAR-T) therapy brings this ethical question into sharp focus [4]. It is not settled by patients’ consent. Many terminally ill people and their families desperately cling to the hope that a novel, experimental drug provides. They value fighting to stay alive more than safety. On the other hand, regulators often prefer a cautious approach to first-in-human trials. Whether a drug is ready for first-in-human use depends on whether you ask a dying patient, a treating clinician, a drug company’s CEO, or a Food and Drug Administration official. Debating what role animal data ought to play in these decisions remains key to their resolution. Ethics research clarifies the values in play and develops arguments favoring one approach over the other—workable solutions subject to criticism and revision.

Some maintain bioethics has done nothing good for science. They see ethical questions as obstacles to scientific progress and national conversations as a drag on innovation. Recent calls for bioethics to “get out of the way” of research heatedly express this sentiment [5,6]. However, such calls reflect a deep misunderstanding of the bioethics enterprise and its impact. Bioethicists are not the science “police.” The field does not exist simply to block innovation or to create boxes that need to be checked by hordes of bureaucrats.

The questions that consume ethicists are of real-world and real-time importance to scientific researchers and lay people alike. It’s not only (and not even usually) PhDs in philosophy who think about whether it’s OK to use animals in research, how to study gene drives in the wild, or when to move from animals to first-in-human use. Looking at those practices, coming up with a vocabulary for talking about the values behind them, and having a dialogue about them not just in ethics journals but also in the media, in classrooms, at the watercooler, and elsewhere are essential for advancing science without engendering misguided or ill-informed pushback. Genome editing and other biomedical and scientific innovations ought not founder simply because a distrustful public has been shut out of discussions, because scientists failed to make an ethical case for moving forward, or because bioethicists were denied the opportunity to create reasonable proposals that accommodate both concern and enthusiasm.

Bioethics pushes scientists to acknowledge that they operate not within a vacuum but within a society in which diverse perspectives and values must be engaged. Bioethicists give voice to those divergent perspectives and provide a framework to facilitate informed and inclusive discussions that spur progress, rather than stall it. In an era in which so many pressing challenges will depend on the innovations of science, from emerging infectious diseases to food security, the need for progress that’s both ethical and accountable has never been greater.

**Image 1:**
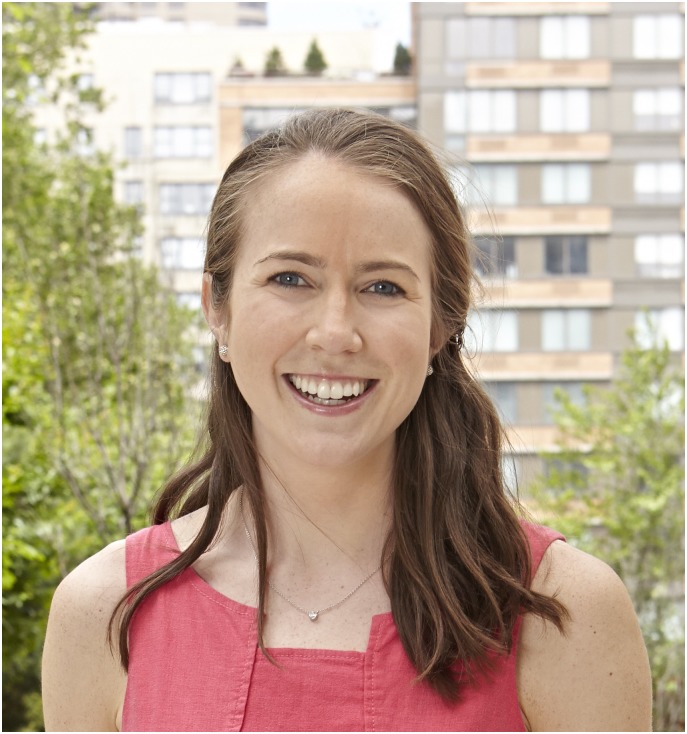
Carolyn P. Neuhaus.

**Image 2:**
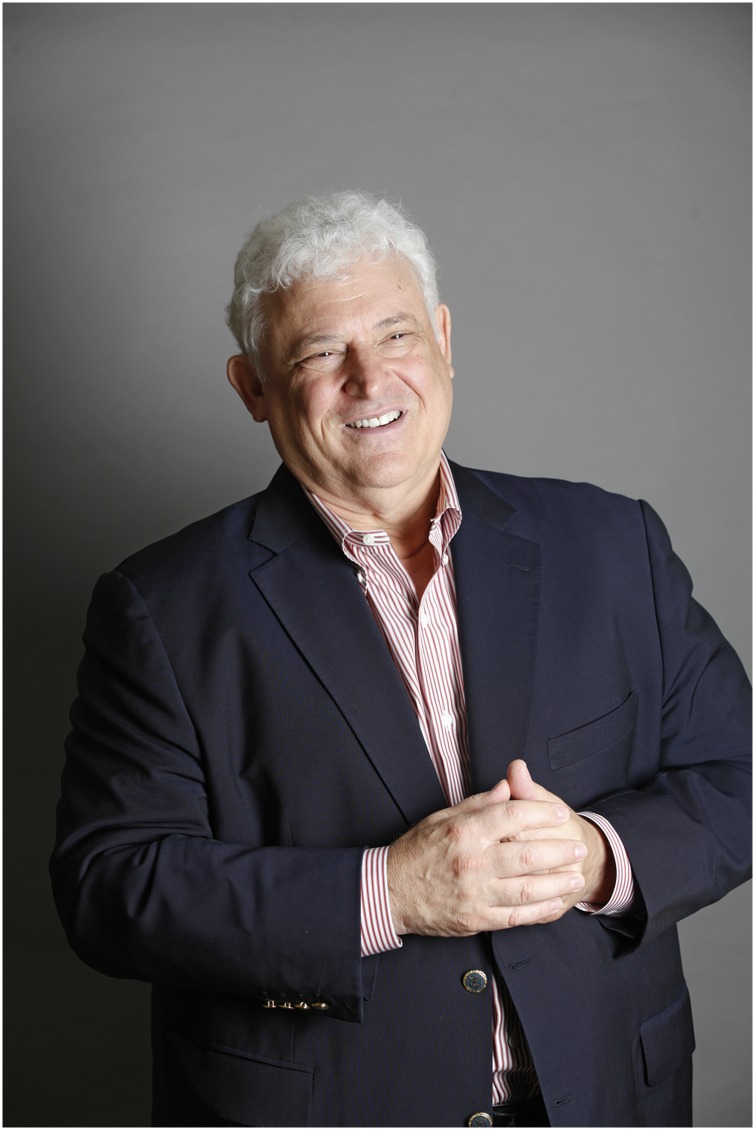
Arthur L. Caplan.
